# The role of pulmonary CT scans for children during the COVID-19 pandemic

**DOI:** 10.1186/s12916-020-01647-1

**Published:** 2020-06-05

**Authors:** Ian P. Sinha, Musa Kaleem

**Affiliations:** 1 Alder Hey Children’s Hospital, Liverpool, L12 2AP UK; 2 Division of Child Health, University of Liverpool, Liverpool, UK

**Keywords:** COVID-19, Imaging, Children, Diagnosis

## Background

It is now well established that children are significantly less likely to develop COVID-19 than adults and that they rarely suffer from severe infection [1]. One area of uncertainty, however, is how best to diagnose children with COVID-19. The accuracy of reverse transcriptase polymerase chain reaction (RT-PCR) is variable and depends on the type of sample taken, the technique used, and when during an infection it is tested [2, 3]. There is an increasing body of literature supporting the use of high-resolution computed tomography (HRCT) scanning for adults suspected of having COVID-19 in selected situations [4], particularly those with severe illness [5].

## What is the role of CT imaging in children with COVID-19?

The linked paper brings useful insight into the role of CT for children with COVID-19 and raises important questions [6]. This retrospective, single-centre study from Wuhan describes the use of routine chest CT scanning in 158 children 16 years old and below who were considered high risk of having COVID-19 based on family or social contact with someone diagnosed with COVID-19. Of 158 children with possible or suspected COVID-19, clinically, 76 had positive RT-PCR and/or at least one change on CT that could be compatible with COVID-19 (43/76 [57%] had positive PCR and positive CT; 7/76 [9%] had positive RT-PCR but no change on CT; 26/76 [34%] had changes on CT, but negative RT-PCR).

Importantly, the article highlights CT abnormalities which can be found in the majority of children with confirmed COVID-19. In the 43 children who tested positive on RT-PCR and had changes on CT, some radiological trends were observed, and these are similar to changes described in adult chest CT scans. The commonest abnormality was ground-glass opacity, which occurred in 29/43 (67%), but perhaps more useful is the location of lesions: changes were seen in subpleural regions in 41/43 (95%) and in the lower lobes in 28/43 (65%). The other important finding was that there was a lag in resolution of CT changes as compared with clinical improvement. This is relevant because, at the time the study was conducted, discharge criteria in China included radiological improvement.

So where does this leave the role of CT in children? The answer is that a ‘one-size fits all’ approach is unlikely to be helpful. For a test to be useful and worthwhile for a patient, at a particular point in time, it should have good diagnostic accuracy, be safe, and have some bearing on clinical decisions.

For children with proven COVID-19 infection but minimal or no respiratory symptoms, we would suggest that chest CT is unlikely to be helpful. In adult COVID-19, which at the time of writing carries a crude in-hospital mortality rate of 30% in the UK [7], understanding the underlying lung pathology may be useful in determining prognosis or identifying appropriate strategies for respiratory support. In most children with COVID-19, who have self-limiting illness, and mild disease progression, knowing the changes on CT is unlikely to guide clinical decisions. For most children with suspected COVID-19, but negative testing, chest CT is also unlikely to be of benefit. If they have mild, self-resolving symptoms, then making the diagnosis is not crucial, and CT (as always) is not a perfect tool for making this diagnosis anyway—as seen in the linked study by Ma et el., nearly 10% of children had positive RT-PCR but negative CT [6].

There are particular children in whom CT scanning of the chest may be useful, depending on their clinical status. CT imaging may be useful for children with signs of hyperinflammation. This might reflect an emerging hyperinflammatory phenotype, which bears some resemblance to Kawasaki syndrome that may, at least temporally, be related to the COVID-19 pandemic [8]. In those selected cases, there is an emergent role of CT coronary angiography. CT imaging may be useful in cases of children with suspected ARDS, or hypoxaemia that is disproportionate to the child’s dyspnoea. In such cases, CTPA (CT pulmonary angiography) may also be warranted to exclude the possibility of associated pulmonary embolism. In children who are unwell with COVID-19, antiviral medication or targeted immune modulation with anti-interleukin 1 or anti-interleukin 6 therapies may be warranted, preferably within the context of a clinical trial.

When considering the use of diagnostic HRCT scanning for COVID-19 in children, additional factors should be taken into account. Paediatric pulmonary CT scans have been associated with an increased lifetime radiation risk and malignancy [9]. There are logistical difficulties in transporting children to a CT scanner, and the process utilises cleaning and safety equipment that in many settings is in short supply.

## Conclusion

Whilst HRCT changes in children with COVID-19 may appear early in the disease process, we should be circumspect about the routine use of HRCT as a diagnostic modality. There may be a role for this test in some children, but as with all investigations, decisions about when to conduct a CT should be made on a case-by-case basis. One size does not fit all.

## Data Availability

Not applicable

## Abbreviations

RT-PCR: Reverse transcriptase polymerase chain reaction
HRCT: High-resolution computed tomography
